# Wavefront Selective Modal Excitations for Optimally Informative Sensing in Fano‐Resonant Metasurfaces

**DOI:** 10.1002/nap2.70119

**Published:** 2026-05-09

**Authors:** Nick Feldman, Nelson de Gaay Fortman, Arie J. den Boef, Lyubov V. Amitonova, A. Femius Koenderink

**Affiliations:** ^1^ Department of Information in Matter and Center for Nanophotonics AMOLF Amsterdam the Netherlands; ^2^ Advanced Research Center for Nanolithography (ARCNL) Amsterdam the Netherlands; ^3^ Department of Physics and Astronomy, and LaserLaB Vrije Universiteit Amsterdam the Netherlands; ^4^ ASML Netherlands B.V. Veldhoven the Netherlands

## Abstract

Fano‐resonant metasurfaces governed by quasi‐bound states in the continuum have recently sparked an enormous interest in the sensing community due to their spectacular sensitivity to nanoscale disturbances. Here, a plane wave or focused illumination is typically used to excite the quasi‐BIC modes. In this paper, we theoretically demonstrate how structuring an incident excitation leads to a gain in information about relevant nanoscale perturbations to be detected. We analyze the full eigenmode spectrum of a dielectric quasi‐BIC metasurface, and uncover eigenmodes that are incompatible with trivial illuminations such as plane waves. By utilizing the concept of “maximum information states,” that was recently developed by the wavefront shaping community, we engineer incident excitations that can selectively excite these quasi‐BIC modes. By using information theory, we quantitatively demonstrate that such wavefront selective modal excitations can be more informative than conventional excitations based on plane waves.

## Graphical abstract

Structured illuminations can unlock hidden quasi‐BIC modes in Fano‐resonant metasurfaces, enhancing the sensitivity to nanoscale perturbations. By analyzing the eigenmode spectrum of a dielectric metasurface and applying information theory, tailored wavefronts can be engineered that selectively excite otherwise inaccessible modes. These selective excitations can outperform conventional plane‐wave excitations in sensing contexts, enabling detection that is more informative and advancing metasurface‐based sensing strategies.

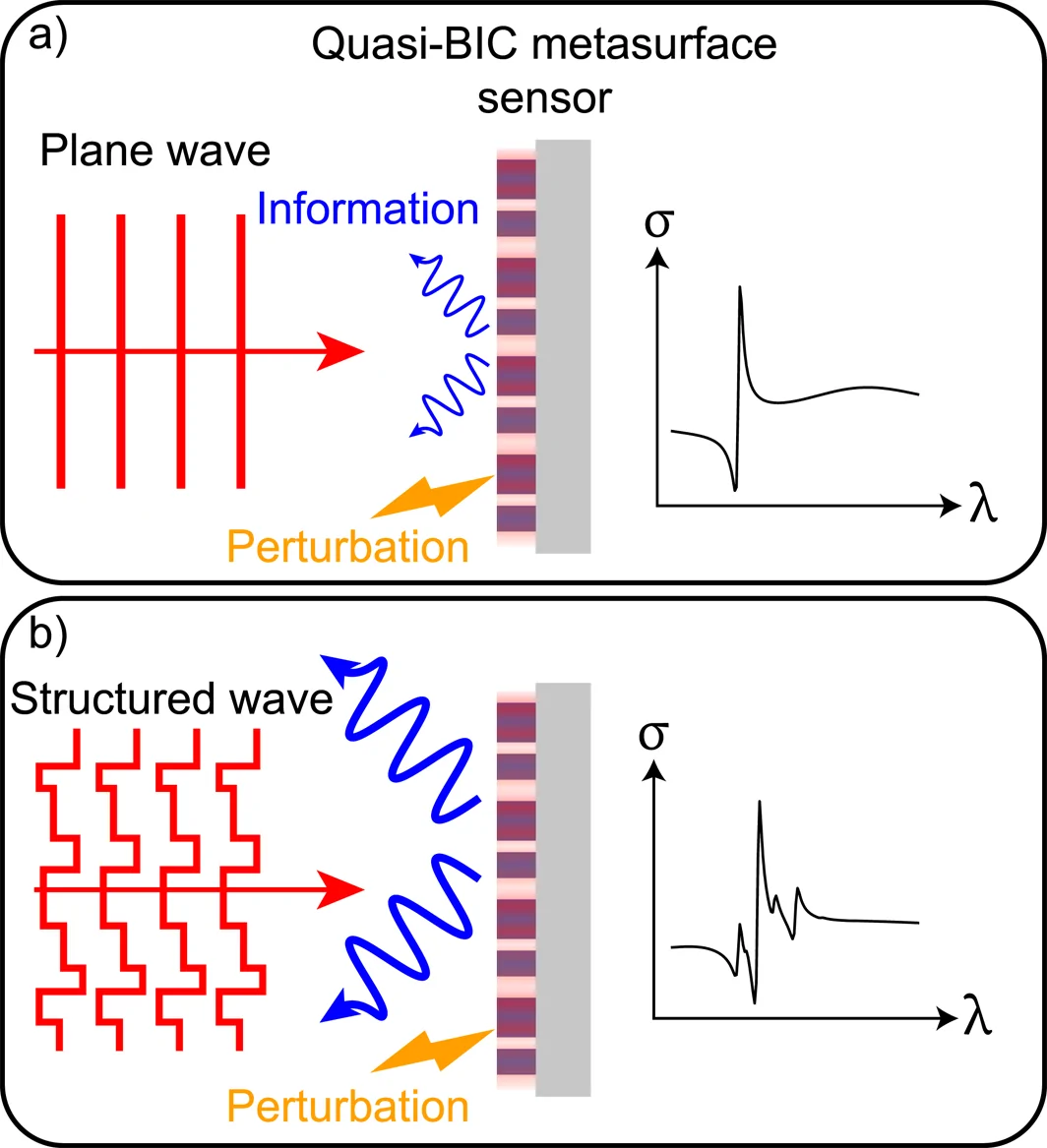

## 1 Introduction

Optical sensing platforms form the backbone of many state‐of‐the‐art metrology applications, ranging from gravitational wave detection [1], wearable sensors for health monitoring [2, 3] and environmental quality control [4]. Here, light is used as a probe that interacts with a physical system, and is subsequently used to read out relevant fundamental parameters of that system. In these applications, an efficient interaction between the incident probe light and the object to be sensed is vital to obtain an informative measurement. This is a highly nontrivial prerequisite in the realm of nano‐optical sensing, where the goal is to retrieve information about specimens on deeply sub‐wavelength length scales. In this regime, the intrinsic light–matter interaction strengths are often insufficient to generate a responsive and detectable sensing signal. Advances in several nanofabrication techniques have allowed to engineer structures that can manipulate incident probe fields that drastically enhance light–matter interactions on the nanoscale. For example, single metallic [5, 6] and dielectric [7, 8] nanoparticles—whose response is dictated by the resonant oscillation of free and bound electrons, respectively—have been shown to efficiently transduce propagating far‐field radiation into locally enhanced near‐field hotspots. These nano antennas [6] have already improved the response in various nanoscale sensing experiments related to, among others, the detection of fluorescence [9], surface‐enhanced Raman scattering (SERS) [10, 11], photo thermal effects [12] and nonlinear signals [13, 14].

Even greater flexibility in shaping the local electromagnetic response of a system can be achieved with the use of metasurfaces [15, 16], which are 2 dimensional sheets decorated by a collection of nanoparticles precisely tailored in size and shape. A powerful advantage of metasurfaces is that they can host strong collective and nonlocal effects between the nanoparticles, offering an enhanced sensitivity in sensing contexts. One of these collective effects is the so‐called bound state in the continuum (BIC) mode [17, 18], which is a spatially extended and resonant state with an infinite quality factor (Q‐factor) embedded within, yet completely decoupled from, the radiation continuum. A true BIC mode cannot be excited by far‐field radiation, and in practical realizations a BIC is transformed into a quasi‐BIC mode with a tunable Q‐factor by introducing a structural symmetry breaking within the metasurface that opens up leakage channels to the far‐field. Quasi‐BIC mode excitation manifests itself as a sharp Fano lineshape [17] in far‐field spectra such as scattering or reflection spectra, and leads to enhanced local fields within the plane of the metasurface. Indeed, recent works have shown that these properties render quasi‐BIC metasurfaces as sensors with spectacular sensitivity to nanoscale refractive index changes in the form of biomolecules [19, 20, 21].

Although nanoparticles and metasurfaces have fostered great innovations in sensing applications by shaping the local near fields at the nanoscale, recent advances in the field of wavefront shaping [22, 23] allow for the flexible tailoring of far‐field propagating modes of radiation with the use of spatial light modulators (SLMs) and digital mirror devices (DMDs). The central challenge of this field is to optimize the response of a complex environment, that usually cannot be engineered in itself, by supplying an external structured far‐field excitation that optimally interacts with the environment. Albeit originally utilized to improve focusing [24, 25] and imaging [26, 27] capabilities through opaque media, wavefront shaping has recently also been exploited to optimally sense parameters in highly complex and disordered environments by maximizing the Fisher information generated by these parameters [28, 29, 30]. Furthermore, by specifically shaping the angular spectrum of an optical field, deeply sub‐wavelength features termed “super oscillations” can be created in the excitation that surpass the diffraction limit [31], which have already been exploited in applications such as super resolution imaging [32] and metrology [33].

Although the domains of quasi‐BIC metasurfaces and wavefront shaping show great promise in enhancing sensing performances by respective structuring of the underlying environments and excitation schemes, combining concepts from both fields to provide a structured excitation that optimally interacts with a quasi‐BIC mode could pave the way toward optimal nanoscale sensor designs. It has recently been demonstrated that quasi‐BIC mode excitation is governed by strict selection rules derived from the symmetry properties of the metasurface [34], analogous to the well‐known selection rules in traditional molecular spectroscopy [35]. These selection rules thereby set specific conditions on the far‐field illumination schemes required to excite the quasi‐BIC modes. In fact, by exploiting very specific structural symmetry features, quasi‐BIC modes can be engineered that are very selective to particularly structured incident wavefronts [36]. These works highlight the importance of structuring the excitation in efficient quasi‐BIC mode excitation. However, the impact of combining optimally structured far‐field excitations with a resonant quasi‐BIC metasurface on nanoscale sensing experiments, and how such excitations should be designed based on the fundamental modes of the meta system, have hitherto not been explored.

In this paper, we theoretically study optimally structured complex excitation modalities in combination with dielectric quasi‐BIC metasurfaces. We find that structured illuminations can lead to wavefront selective modal excitations that are incompatible with conventional illumination schemes, such as plane wave illuminations, and that these selective excitations could offer an enhanced performance in a sensing context, as conceptually depicted in Figure 1. To retrieve these optimally structured excitations, we utilize the concept of “maximum information states” [28, 30] recently developed by the wavefront shaping community for precise parameter estimations in disordered systems, and expand these states into the realm of structured meta systems. Finally, by using information theory, we quantitatively determine how much information a structured excitation generates about a relevant nanoscale parameter to be sensed.

**FIGURE 1:**
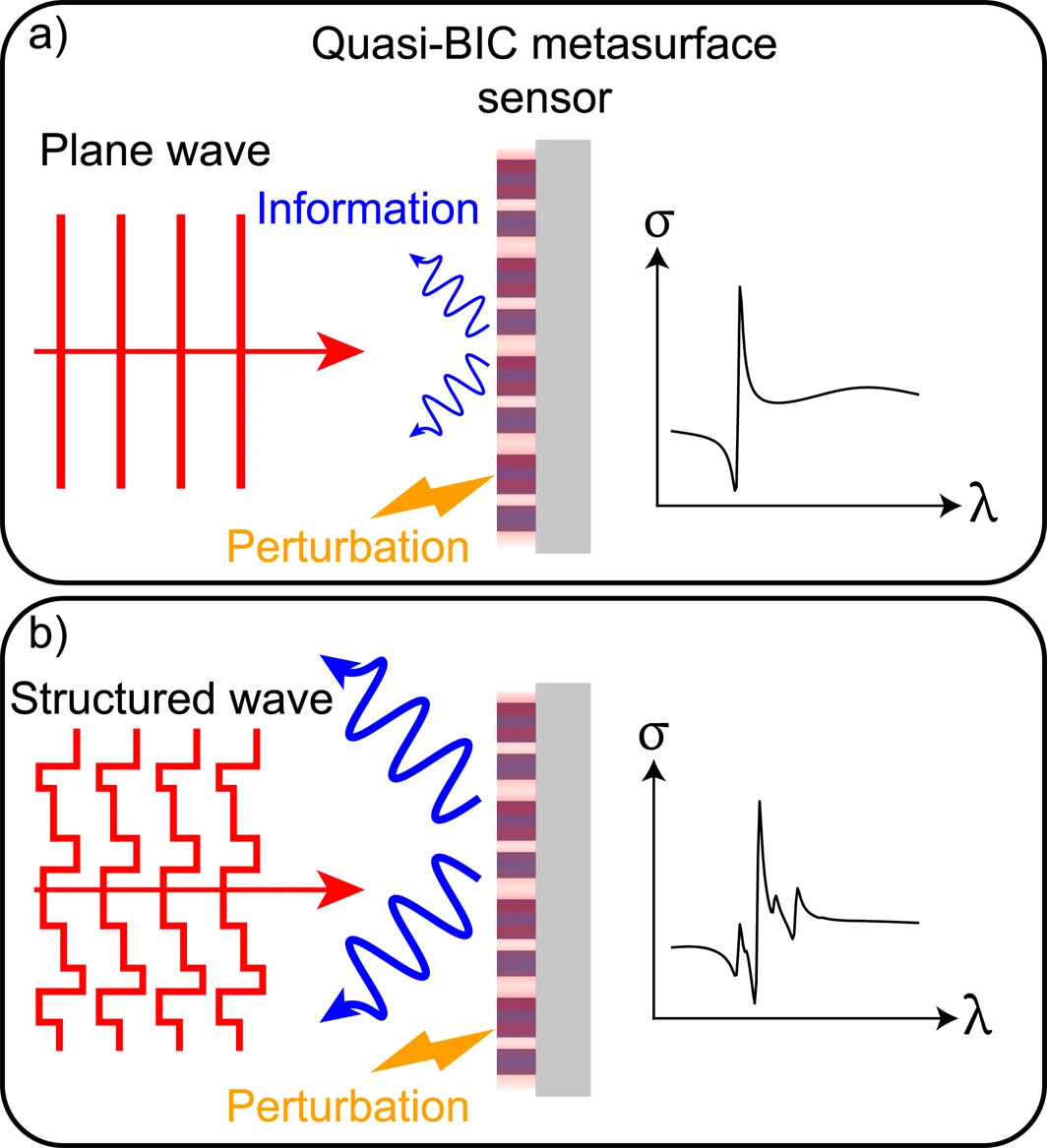
Quasi‐BIC metasurface sensing with structured excitations. (a) A conventional metasurface quasi‐BIC mode is excited by a plane wave. The sensitivity of the quasi‐BIC mode generates information about a nanoscale perturbation, which can be read out in the far‐field. (b) A structured wave selectively excites quasi‐BIC modes that are incompatible with plane wave illuminations, providing an increase in information about the perturbation.

## 2 Metaring Eigenmodes

The resonant meta system that we will theoretically study in this work is depicted in Figure 2a. The meta system is composed of dielectric silicon nanorods positioned in a ring, in which the long axis of the rods is aligned parallel to the radial direction of the ring. A recent study [37] has shown that these meta rings support symmetry protected radial BIC modes, which can be excited by far‐field radiation and turned into quasi‐BIC modes by introducing a structural asymmetry within the nanorods of the meta ring. Specifically, this asymmetry corresponds to a length mismatch between every neighboring rod in the ring ΔL, as indicated in Figure 2a. Quasi‐BIC mode excitation manifests in the form of a sharp Fano lineshape in observables such as scattering spectra, and the enhanced sensitivity at the resonance condition can then be exploited to sense perturbations on the nanoscale. For the sake of concreteness, in this work we focus on a specific perturbation in the meta ring, and make a choice that is inspired by problems that are encountered in state‐of‐the‐art semiconductor chip manufacturing processes [38, 39], where misalignments down to the sub‐nanometer scale can already lead to malfunctioning chips. The resonant meta rings could thus be exploited as telltale scattering sensors that report on the strength of the misalignment to be monitored. The specific perturbation relevant to this paper is defined in Figure 2b, and corresponds to an upward shift of every other nanorod in the ring by Δy. Such perturbations, for instance, occur in microchip manufacturing when devices are “interlaced”, that is, placed in the same sample layer using different lithography exposure steps. We note that the results of this work are by no means restricted to this specific form of perturbation and may also be applied to sense other forms of nanoscale disturbances, such as monitoring environmental refractive index changes or the presence of molecular species.

**FIGURE 2:**
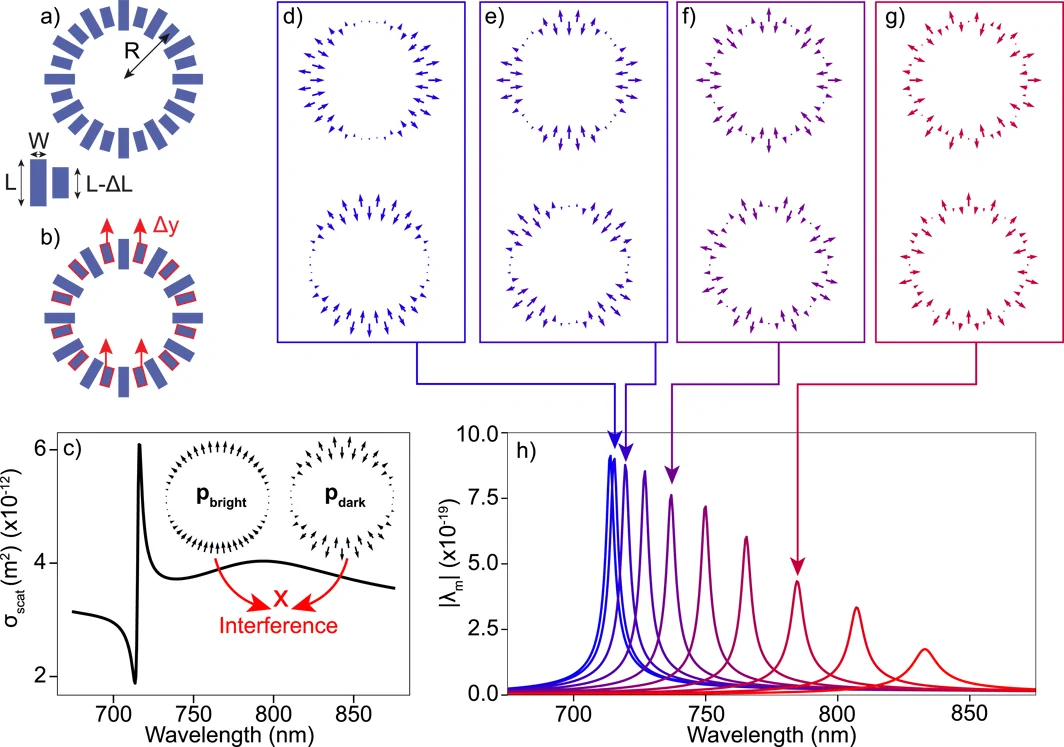
Eigenmodes of the metasurface scattering sensor. Panels (a) and (b) denote unperturbed respectively perturbed versions of the resonant metasurface scattering sensor. The perturbation corresponds to an upwards shift of every other meta atom in the ring by Δy. (c) Scattering cross section of the meta ring for a y‐polarized plane wave excitation, normally incident onto the ring. The inset depicts the dominant eigenmodes responsible for the Fano interference. (d–g) Exemplary dipole configurations of the dominant eigenmodes in the meta ring. (h) Absolute value of the eigenvalue versus wavelength of a series of dominant eigenmodes. The arrows indicate which eigenmode profiles correspond to which eigenvalue spectrum.

To theoretically describe the response of the meta rings, we make use of a home‐built coupled dipole model, of which the theoretical principles are laid out in the Methods section. In short, the individual nanorods within the meta ring are approximated by dipolar point particles described by a polarizability tensor ${\overset{↔}{\alpha }}_{i}$. By using the knowledge of the positions and polarizability tensors of all participating particles, an interaction matrix M can be constructed, which describes the physics of all dipole–dipole coupling mechanisms. Upon excitation by an external driving field $E_{ext}$, the collection of particles is excited and an ensemble of coupled dipoles $p_{i}$ can be calculated by inverting the interaction matrix M. Here all multiple scattering events between the particles are self‐consistently taken into account. We model the individual nanorods by prolate ellipsoids with major axis L=240 nm and minor axis W=100 nm, which are described by an analytically known polarizability tensor [40]. In the following calculations, the radius R of the meta ring is set to R=1.5μm, the number of particles N in the ring is N=48, ΔL=50 nm and Δy=1 nm unless otherwise stated.

The whole notion of exciting a quasi‐BIC mode for sensing requires a sufficient spatial overlap between the external driving field $E_{ext}$ and the relevant fundamental mode at play. To start the discussion, let us recuperate some well‐known facts by considering, for instance, excitation by a y‐polarized plane wave normally incident onto the meta ring. Figure 2c shows the resulting scattering cross section of the meta ring using this illumination condition. A single Fano lineshape is evident, which can be described by a 2 mode interference effect between a super‐radiant “bright” mode $p_{bright}$, that is, a mode that can radiate efficiently into the far‐field, and a sub‐radiant “dark” mode $p_{dark}$, with an inhibited coupling to the far‐field. This is the well‐known Fano resonance in photonics [41]. The two relevant modes are visualized in the inset of Figure 2c, where both $p_{bright}$ and $p_{dark}$ have a nonzero overlap with $E_{ext}$. In the case of $p_{bright}$ this is evident, since $p_{bright}$ and $E_{ext}$ are both odd in parity. By virtue of the asymmetry ΔL, a small net dipole moment is present in the even parity $p_{dark}$, which therefore generates an overlap with $E_{ext}$. To obtain the highest amount of information about the perturbation Δy in a sensing experiment, the wavelength can now be tuned to resonance (approximately 710 nm) to optimally make use of the high sensitivity of the quasi‐BIC mode.

**FIGURE 3:**
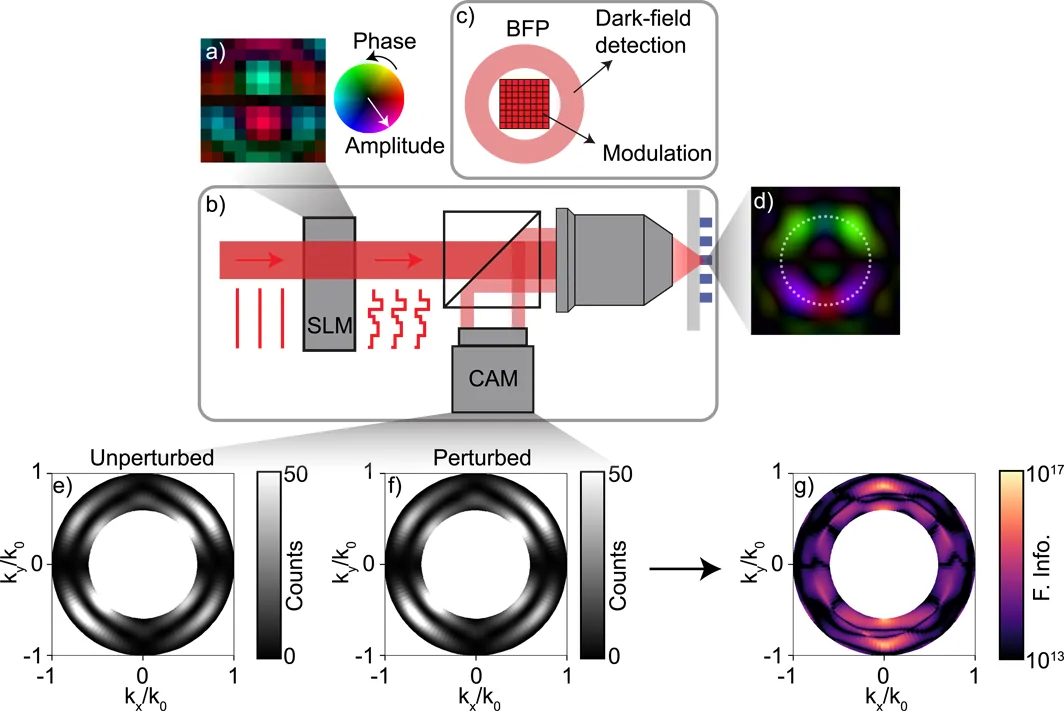
Numerical wavefront shaping pipeline. (a) Complex modulation map corresponding to a modulated incident wavefront. Complex values are represented by a color wheel, where the hue indicates the phase, and the brightness indicates the amplitude of the wavefront. (b) Cartoon of the numerical wavefront shaping setup. The SLM indicates complex modulation of an incident wavefront, the objective focuses the incident wavefront onto the sample plane, after which a camera detects the far‐field scattered intensity originating from the sample (c) Schematic of the illumination and detection modes in the calculations, mimicking dark‐field detection. (d) Complex excitation map in real space corresponding to the modulation of panel (a). The gray circles indicate the positions of the dipoles in the meta ring, and the scalebar corresponds to 1 μm. (e) and (f) Scattered far‐field intensity images of an unperturbed and perturbed meta ring. (g) Far‐field Fisher information map corresponding the signals of panels (e) and (f).

A surprising observation is that the established $p_{bright}$ and $p_{dark}$ that are responsible for the commonly observed Fano resonance under normal incidence are in fact neither the only eigenmodes of the system, nor the only high‐Q BICs. To gain a complete picture of all the eigenmodes in the meta ring, we perform an eigenvalue decomposition on the interaction matrix M, from which we can extract all relevant eigenmode profiles ${\tilde{p}}_{m}$ and corresponding eigenvalues $\lambda_{m}$. A selection of the eigenmodes with the highest eigenvalues is visualized in Figure 2d–g, along with the magnitude spectrum of the corresponding eigenvalues $\lambda_{m}$ in Figure 2h. These eigenvalues represent how strongly each collective eigenmode ${\tilde{p}}_{m}$ can be excited by an external field $E_{ext}$, provided there is sufficient overlap between ${\tilde{p}}_{m}$ and $E_{ext}$, and can be regarded as modal polarizabilities. The complete set of eigenmodes corresponding to all eigenvalue spectra is shown in the Supporting Information S1. It can be seen that the retrieved eigenmodes come in degenerate pairs, which are mutually rotated, highlighting the high symmetry conditions of an unperturbed meta ring. Except for the dark mode that we have already encountered in Figure 2c, Figure 2e–g also depict other types of even parity eigenmodes with a seemingly increasing envelope modulation frequency along the circumference of the meta ring. This spatial change in eigenmode shape leads to a corresponding redshift in eigenmode resonance frequency, as can be observed from the spectra in Figure 2h. This phenomenon is analogous to the well‐known concept of mode hybridization in plasmonic systems [42], wherein coupled dipole modes experience energy shifts depending on the relative dipole orientation, a phenomenon likened to molecular orbital hybridization. Similar effects can occur with coupled dipole resonances in dielectric systems as well. A crucial difference between the modes in Figure 2e–g from the dark modes shown in Figure 2c,d is that the former modes have *zero* overlap with a plane wave excitation. This is the fundamental reason why these eigenmodes do not manifest as Fano lineshapes in the scattering spectrum of Figure 2c. However, from the eigenvalue spectra in Figure 2h it can be seen that these modes possess all the desirable properties of quasi‐BIC modes, such as tunable high Q‐factor resonances accompanied by strong polarizabilities, and could thereby be exploited as potentially informative excitations to probe Δy. We note that such modes, incompatible with plane wave excitations, are not necessarily unique to this specific meta ring geometry and might also be found in other types of nanophotonic structures, such as metasurfaces composed of periodic square lattices, oligomers of nanoparticles and even single nanoparticles [43]. The key questions we would like to tackle in the following sections is how these modes could be addressed by supplying a designer structured excitation $E_{ext}$ with a nonzero spatial modal overlap, and to quantify how much information about the perturbation can be gained by such selective modal excitations.

## 3 Wavefront Shaping Pipeline

The external degrees of freedom offered by wavefront shaping offer an ideal toolbox to suitably structure the incident field and to offer the necessary modal overlap to excite the modes discussed in the previous section. To this end, we have developed a numerical wavefront shaping pipeline, which is schematically visualized in Figure 3. We imagine a scenario in which a meta ring is interrogated in a Fourier microscope, with a liquid crystal spatial light modulator (SLM) in the excitation path, as sketched in Figure 3b. In the calculations, we modulate the amplitude and phase of an incident wavefront within a single polarization channel (y‐polarization), as SLMs typically operate just for one polarization channel. Figure 3a shows an exemplary complex modulation map encoded onto an incident wavefront, where complex values are represented by a color wheel. Here, the hue is connected to the phase, and the brightness is connected to the amplitude. This color wheel visualization is used to represent complex fields throughout the remainder of the paper. Individual pixels in the incident wavefront correspond to individual plane waves with different in‐plane wave vector components, which are subsequently focused onto the sample consisting of the dielectric meta ring scattering targets. Figure 3d shows the y‐component of the corresponding complex structured excitation field in real space relative to the dipole positions in the meta ring, as indicated by the gray circles. Due to focusing, the full vectorial excitation field will also contain components along the *x*‐ and *z*‐directions, but these components are an order of magnitude smaller in amplitude than the y‐polarized component. This excitation field excites dipole moments in the dielectric meta ring, which are governed by the eigenmodes discussed in Figure 2, and the scattered far‐fields of these dipole moments are subsequently calculated and converted into far‐field angle resolved intensity images $I(\hat{k})$, which will be the relevant signal to read out perturbations. In order to mimic a realistic experimental dark‐field scattering detection scheme, we utilize a low numerical aperture (NA) illumination, while we calculate the scattered fields in a high NA region that falls outside the low NA illumination region, as shown in Figure 3c. The recorded dark‐field intensity images are shown in Figure 3e,f, which correspond to the scattered far‐field signals of an unperturbed $I_{0}(\hat{k})$ (Δy=0 nm) and perturbed $I_{\Delta y}(\hat{k})$ (Δy=1 nm) ring, respectively.

To determine how much information a specific calculated far field signal contains about the perturbation at hand, we calculate the Fisher information content of the far‐field scattered signal. Suppose we have access to a dataset X, which in an experimental setting will be unavoidably corrupted by noise, and which in our case corresponds to the far‐field scattered intensity patterns of Figure 3e,f. The Fisher information F relates to the best sensing precision $\sigma_{\theta }$ with which perturbations θ can be estimated from inherently noisy data X according to the Cramer–Rao bound

(1) $$\sigma_{\theta }\geq \frac{1}{\sqrt{F}}$$

and is defined as [44].

(2) $$F=E\left[{\left(\frac{\partial \;\ln\;p\left(X;\hat{\Theta }\right)}{\partial \hat{\Theta }}\right)}^{2}\right],$$

where $p\left(X;\hat{\Theta }\right)$ denotes the probability of measuring a dataset X given a perturbation strength $\hat{\theta }$, and E[.] is the expectation operator. We assume that shot‐noise is the dominant noise contribution in a potential experimental setting. The probability density function $p\left(X;\hat{\Theta }\right)$ is then described by a Poisson distribution, and it can be shown that the Fisher information is described by the following expression:

(3) $$F(\hat{k})=\frac{1}{I(\hat{k})}{\left(\frac{\partial I(\hat{k})}{\partial \hat{\Theta }}\right)}^{2}.$$

If the derivative in Equation (3) is approximated by finite differences, Equation (3) emphasizes the fact that a pixel wise far‐field Fisher information pattern $F(\hat{k})$ can be constructed by using both unperturbed and perturbed far‐field patterns $I(\hat{k})$. The far‐field Fisher information pattern corresponding to the unperturbed and perturbed intensity images of Figure 3e,f is shown in Figure 3g. This pattern reveals how much information is scattered into specific far‐field detection angles $\hat{k}$. Since Fisher information is additive, the total Fisher information of the calculation can be determined by simply summing all values in the Fisher information pattern $F(\hat{k})$. Using this information theoretical formalism, we can now quantify how informative each individual incident wavefront is, and it is our objective to find the wavefront that maximizes the total Fisher information to sense perturbations Δy.

The problem of finding these so‐called maximum information states has already been tackled in several works, for instance by using nonlinear optimization algorithms [29, 45, 46, 47] or by determination of the (partial) scattering matrices of the system [28, 30]. In this work, we utilize the algorithm proposed in [28], which retrieves the illumination that maximizes the Fisher information assuming intensity‐only signals. In brief, the algorithm exploits higher order tensors, which are constructed from the partial scattering matrices of the sample under study, and provides a convenient solution based on linear algebra even though the dependence between the calculated far‐field intensities and the excitation fields is quadratic. We thereby determine the partial scattering matrix of a meta ring scattering target $H_{ij}$, along with its derivative with respect to perturbations $\partial_{\theta }H_{ij}$, and construct the higher order tensor $W_{\mathrm{ijk}}=\partial_{\theta }(H_{ij}^{*}H_{ik})$. The wavefront that maximizes the Fisher information can then be found by performing a best rank‐one approximation of $W_{\mathrm{ijk}}$. The complex pattern shown in Figure 3a is the resulting wavefront after applying this tensor based optimization scheme at a wavelength of 710 nm. At this wavelength, the modes shown in Figure 2c,d are the most dominant in the scattering response of the meta ring. Therefore, one intuitively expects that the optimum illumination provides the best spatial overlap with the dominant mode at play in order to fully exploit the high sensitivity at resonance, and that these maximum information illuminations could be used as a tool to selectively excite modes incompatible with trivial excitation schemes.

## 4 Optimum Illuminations and Selective Modal Excitations

To evaluate whether optimally informative illuminations correlate to selective modal excitations within the meta ring, we calculate optimum wavefronts to sense Δy for a range of different wavelengths covering the spectral regime of Figure 2h. The resulting complex excitation maps in the sample plane are depicted in Figure 4a–c, for wavelengths of 710, 730, and 750 nm, respectively. At these wavelengths, we expect that different eigenmodes will dominate the scattering response according to Figure 2h. For comparison, a more trivial excitation map, corresponding to a softly focused beam at 750 nm, is visualized in Figure 4d. First, it can be seen that the magnitude of all optimally informative excitation fields is strongest at the actual positions of the dipolar scatterers, providing a ring‐shaped illumination pattern. This is logical, since the interaction with the excitation field and the scattering sensor should be maximized to obtain the most informative measurement. Second, strong modulations within the optimum excitations can be discerned, which are highly wavelength dependent. In other words, although in previous work meta rings have mainly been studied as exquisite sensors under plane wave incidence, they can in fact be at least as informative under more complex illuminations. At first glance, these modulations seem to increase along the circumference of the ring for increasing wavelength, similar to the increase in modulations in the eigenmode configurations of Figure 2d–g. This indicates that the excitation is optimized to couple efficiently to the dominant fundamental eigenmode.

**FIGURE 4:**
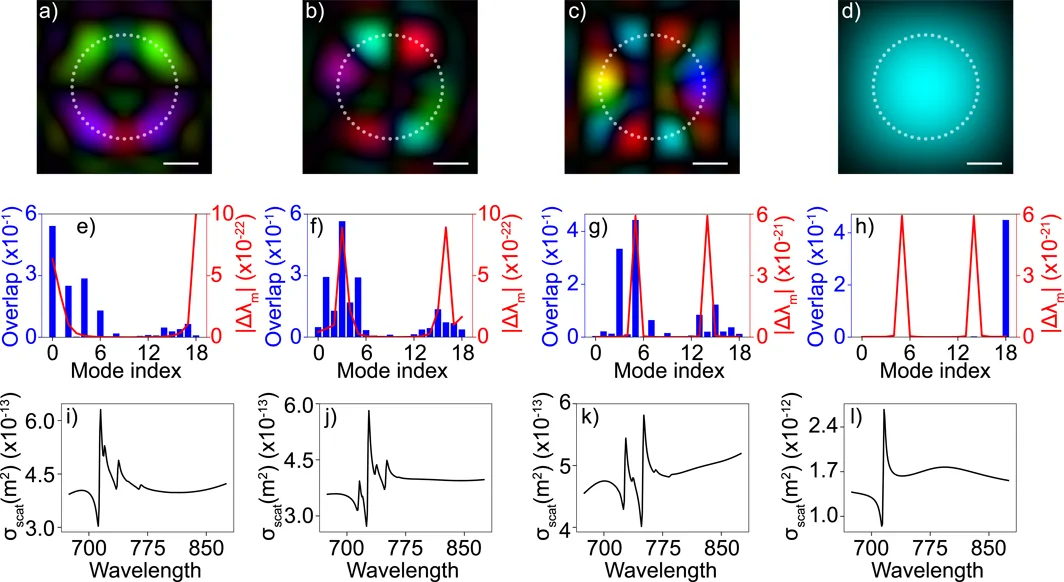
Selective modal excitations in Fano resonant meta rings. Panels (a–c) show complex excitation maps of the optimally informative illumination in the plane of the meta ring for a wavelength of (a) 710 nm, (b) 730 nm and (c) 750 nm. Panel (d) shows the complex excitation map of a softly focused beam at a wavelength of 750 nm. The same color wheel convention to visualize complex fields is used as in Figure 3. (e–h) Spatial modal overlap of the excitations in panels (a–d) with all dominant eigenmodes in the meta rings, indicated by the blue vertical bars. The indexing of the modes follows the same convention as Supporting Information S1: Figure S1. In the same panels, the change in magnitude of the mode eigenvalue due to the perturbation is highlighted by the red solid line at the same wavelengths as panels (a–d). (i–l) Scattering cross sections of an unperturbed meta ring corresponding to the excitations in panels (a–d).

To quantify this statement, we calculate the overlap between the optimum excitation $E_{ext}$ and all relevant eigenmodes ${\tilde{p}}_{m}$ according to $\langle E_{ext}|{\tilde{p}}_{m}\rangle$, and plot the results in Figure 4e–h as blue vertical bars. Here, the mode indexing follows the convention as defined in Supporting Information S1: Figure S1. This optimally structured excitation couples to a plethora of eigenmodes, contrary to the trivial illumination of Figure 4d, which can only efficiently couple to a single mode corresponding to the dark mode of Figure 2c. Moreover, the overlap spectrum changes dramatically for different optimum excitation fields. In the same Figure 4e–h, we plot the change in magnitude of the eigenvalues $\left|\Delta \lambda_{m}\right|$ corresponding to each mode due to the perturbation Δy as red solid lines, again for wavelengths of 710, 730, 750 and 750 nm, respectively. This plot indicates which modes are most sensitive in polarizability for different wavelength regimes, and is symmetric on the *x*‐axis due to the degeneracies present in the eigenmodes. A high correlation between the differential modal eigenvalue spectrum and the modal overlap spectrum of the excitation can be observed. This verifies the hypothesis that optimally informative illuminations selectively enhance their coupling to the most sensitive fundamental modes of the system.

To highlight the effect of these optimally informative illuminations on the scattering response of the meta system, we calculate the scattering cross sections of the meta rings for every individual optimum excitation, and show the results in Figure 4i–l. Instead of a single Fano lineshape that corresponds to the trivial excitations of Figures 4d and 2c, multiple Fano lineshapes are now discerned in the scattering spectrum for the complex structured excitations, which are a direct result of the enhanced modal coupling discussed in Figure 4e–g. Comparing the shape of the individual spectra for different optimum excitations, we can further observe that the relative modulation depth of the Fano lineshapes in the spectrum is highly wavelength dependent, reflecting the enhanced and suppressed coupling to the underlying quasi‐BIC mode. Here, the spectral position of the Fano lineshape with the highest modulation depth corresponds to that spectral region for which the excitation field was optimized for sensing. Finally, the overall modulation depth of the Fano lineshapes decreases for increasing wavelength, primarily consistent with the decreasing polarizability of the dominant eigenmodes as shown in Figure 2h, whereas additional weak multipolar excitation channels may further reduce the spectral contrast by partially smearing the two‐mode interference. These results underscore the potential of structured excitation schemes in combination with quasi‐BIC metasurfaces to uncover modal excitations that are incompatible with conventional (plane wave) illuminations. It should be noted that the structural symmetry breaker ΔL is still crucial here for efficient modal excitation, without which no overlap with any far‐field excitation scheme could be achieved.

## 5 Sensing Precision

We next quantify how much information can be gained from these wavefront selective modal excitations, and compare the information content of an optimally structured excitation with that of more trivial illumination schemes. For this, we calculate the total Fisher information of the numerical sensing experiment according to Equation (3) for three different excitations that are visualized in Figure 5a–c. These excitations correspond to a softly focused beam in Figure 5a, a focused orbital angular momentum (OAM) beam [48] in Figure 5b, which in this case provides a donut‐shaped illumination containing a phase vortex that is compatible with the annular shape of the meta ring, and finally the excitation retrieved from the optimization algorithm in Figure 5c, respectively. For a fair comparison, we fix the incident average intensity for all excitations to $10^{15}$ photons per $m^{2}$. The Fisher information is calculated for the range of wavelengths in which the relevant eigenmodes of the meta ring are dominant, where the optimum excitation is recalculated for each individual wavelength. The Fisher information as a function of wavelength for the three excitation schemes is shown in Figure 5d. It can be observed that the total Fisher information of the optimal excitation is higher than the trivial excitations for all wavelengths, as expected from the optimization algorithm. The OAM excitation seems to be more informative than the focused excitation for a broad range of wavelengths as well. This can be explained by the fact that the OAM excitation has an intensity null exactly in the center of the ring, where no scattering particles are present. We can further see that all three excitations have a strong information peak at 714 nm, reflecting the fact that exciting the most sensitive eigenmode provides the most informative measurement. Even at the fundamental resonance, the optimum excitation outperforms the trivial excitation by a factor of 5 in information content. Besides the main peak at 714 nm, the optimum excitation shows multiple strong information peaks that correspond to the wavefront selective modal excitations, whereas the Fisher information for the trivial illuminations decreases dramatically in this spectral range. As can be seen, these selective excitations radiate a similar information content about the perturbation as the optimum excitation at the fundamental resonance, and selective excitations that address the nontrivial modes can provide even more information than the canonically used Fano resonance in the simple plane–wave excitation manifold.

**FIGURE 5:**
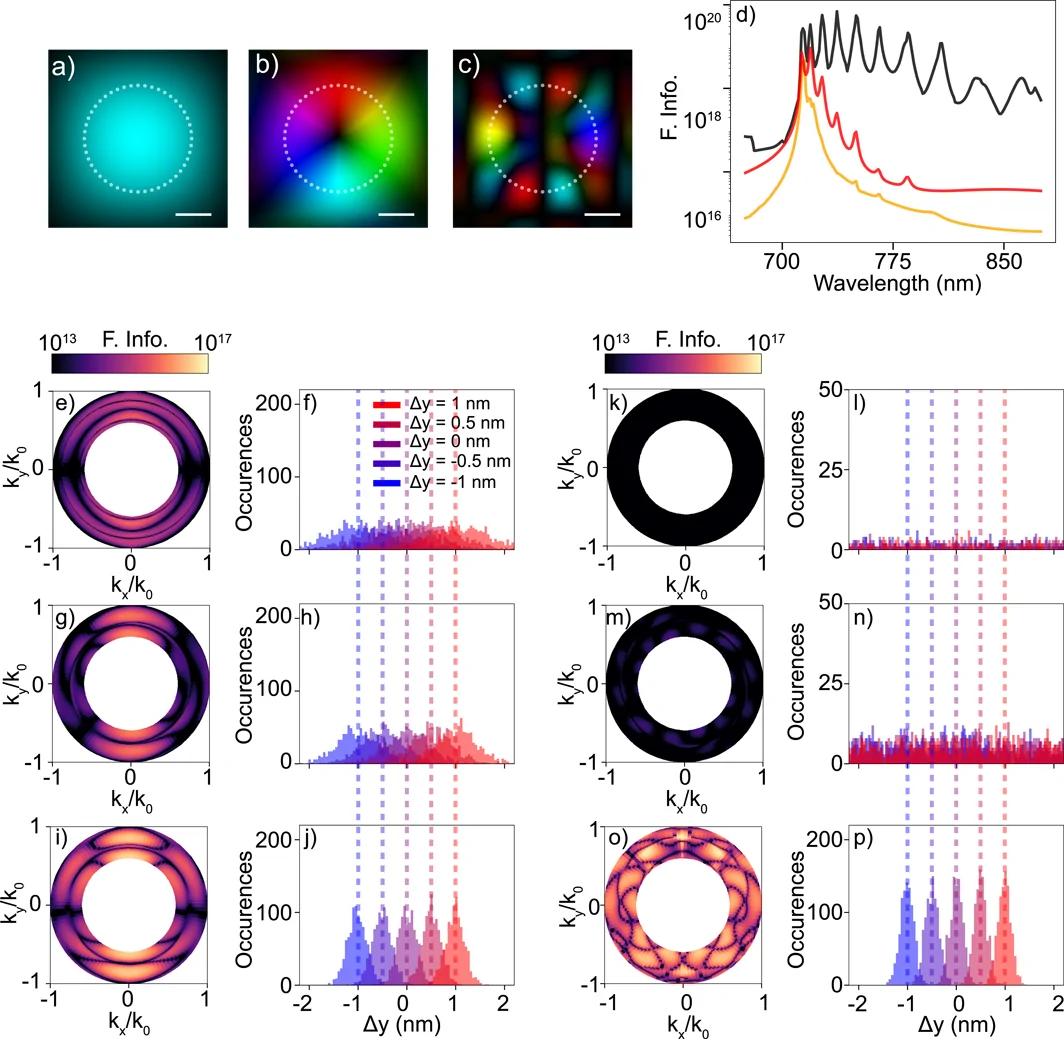
Information quantification and estimation precision. Panels (a–c) visualize complex excitation maps for (a) a softly focused beam, (b) an OAM beam and (c) the optimum excitation. The scalebar corresponds to 1 μm (d) total Fisher information versus wavelength for the excitations of panels (a–c). The black, red and orange line denote the optimum, OAM and softly focused excitations, respectively. Panels (e), (g) and (i) show angle resolved Fisher information patterns of the meta rings for the softly focused, OAM and optimum excitations, respectively, where the excitation wavelength is set to 714 nm. Panels (f), (h) and (j) show estimation histograms of unknown perturbations using the softly focused, OAM and optimum excitations, respectively. (k–p): Same as panels (e–j), but at an excitation wavelength of 750 nm.

We finally describe how these excitations, in combination with the scattered signal of the meta rings, could be used to perform precise estimations of potentially unknown perturbation strengths. For this purpose, it is necessary to define an unbiased estimator, which is an operation that translates noisy measured or calculated data into an estimation of the perturbation $\hat{\theta }$. The estimator $\hat{\theta }$ is termed unbiased if the average of $\hat{\theta }$ over the noise fluctuations equates the true perturbation θ. We assume that we are dealing with small perturbations down to the sub‐nanometer scale, in accordance with modern semiconductor wafer metrology requirements. In this regime of small parameter variations, the far‐field scattered signal $I_{\Delta y}(\hat{k})$ corresponding to a perturbation Δy can be described by a linear model according to:

(4) $$I_{\Delta y}(\hat{k})=I_{0}(\hat{k})+\Delta y\frac{\partial I_{0}(\hat{k})}{\partial \left(\Delta y\right)}+W(\hat{k}),$$

where $I_{0}(\hat{k})$ is the far‐field scattered signal corresponding to an unperturbed meta ring, $\frac{\partial I_{0}\left(\hat{k}\right)}{\partial \Delta y}$ is the change in scattered signal due to an infinitesimal perturbation, evaluated at Δy=0 nm, and $W(\hat{k})$ is a normally distributed vector that approximates the Poisson distributed noise in the signal. For the linear model described by Equation (4), an expression for the unbiased estimator that reaches the Cramer–Rao lower bound can be derived as [44]:

(5) $$\hat{\theta }=\frac{1}{F_{0}}\sum_{i=0}^{p}\frac{\partial I_{0}(\hat{k})}{\partial \left(\Delta y\right)}\left(\frac{I_{\Delta y}(\hat{k})-I_{0}(\hat{k})}{I_{0}(\hat{k})}\right),$$

where $\hat{k_{i}}$ corresponds to the detection angle at pixel i on the detector, p the total number of pixels on the detector, and $F_{0}$ the Fisher information corresponding to the two measurements in the differential $\frac{\partial I_{0}\left(\hat{k}\right)}{\partial \Delta y}$. If we again approximate the differential in Equation (5) by finite differences, it can be seen that an estimation of unknown perturbations Δy can be carried out from a noisy far‐field image $I_{\Delta y}(\hat{k})$ by first conducting a noise free calibration calculation on the scattered signal of an unperturbed meta ring, and of a meta ring at a known, small perturbation strength. In this calibration calculation, we set the known perturbation strength to 0.1 nm. We next calculate far‐field scattered signals of the meta rings for the illumination conditions described in Figure 5a–c at perturbation strengths of Δy=−1 nm, Δy=−0.5 nm, Δy=0 nm, Δy=0.5 nm and Δy=1 nm, and generate 1000 different configurations of these images covered by shot‐noise in order to simulate a sequence of measurements. We then apply Equation (5) to these datasets to gather statistics on the estimation precision.

Figure 5e,g,i show angle resolved Fisher information maps $F_{0}(\hat{k})$ of the calibration calculation for the softly focused, OAM and optimal excitation, respectively, where the excitation wavelength is set to 714 nm. Here, the increase in Fisher information can be recognized when an optimally structured excitation is offered. Figure 5f,h,j report on the corresponding estimation histograms of the noisy datasets under the respective excitation conditions. For the estimations of all datasets, the mean value of the estimation histograms is equal to the true perturbation strength, indicating accurate and unbiased estimations of Δy. For the estimations conducted on the datasets retrieved by the softly focused excitation in Figure 5f, a large overlap between individual histograms is seen, which means that the retrieved estimations are not precise. For the OAM excitation in Figure 5h, the individual distributions become narrower, whereas for the optimum excitation in Figure 5j five sharp peaks with minimum overlap between the histograms are seen, commensurate with a measurement with maximum Fisher information. We repeat this procedure in Figure 5k–p at an excitation wavelength of 750 nm, detuned from the quasi‐BIC mode at 714 nm. Estimating a perturbation at this wavelength using a softly focused or OAM excitation results in practically meaningless histograms from which no precise estimations can be made, which is a consequence of the dramatic decrease in Fisher information using these illuminations. When selectively exciting the underlying quasi‐BIC mode, however, the 5 well‐defined estimation histograms are recovered, with a sensing precision comparable to the optimum excitation at 714 nm.

## 6 Conclusions

In conclusion, we theoretically studied complex, structured excitation modalities in quasi‐BIC metasurface sensors, and showed how such excitations lead to an information gain about the relevant perturbation to be sensed. We mapped the eigenmode spectrum of a dielectric Fano resonant meta ring, and, in addition to the well‐known q‐BIC responsible for the Fano resonance under plane wave incidence, we uncovered a plethora of hitherto unknown quasi‐BIC modes that cannot be directly addressed by conventional plane wave illumination schemes. We showed that optimally structuring an excitation using wavefront shaping allows for the excitation of such quasi‐BIC modes, and that these selective modal excitations could be used as informative responses to probe nanoscale perturbations. We demonstrated that wavefront selective excitations could be even more informative than excitations by conventional illuminations within the same metasurface, prompting the metasurface community to think of the rich wavefront shaping toolbox as an extra degree of freedom to optimize the metasurface response.

Looking ahead, a single structured excitation could be engineered that efficiently couples to multiple quasi‐BIC modes across a broad spectral range, which might be relevant in broadband‐sensing applications. Besides sensing, wavefront selective excitations might also find relevance in the field of nonlinear optics, where high harmonic signals could be selectively enhanced or suppressed depending on the incident wavefront. Instead of structuring an excitation in the spatial domain, state‐of‐the‐art ultrafast pulse shaping methods [49] also allow to engineer the excitation in the temporal domain, which may pave the road toward optimal spatio‐temporal coupling of excitations to quasi‐BIC modes. Overall, our work highlights the untapped potential of wavefront engineering as a powerful lever to unlock the full potential of resonant metasurfaces, laying the groundwork for a new class of ultra‐sensitive, spectrally versatile, and dynamically tunable photonic sensors.

## 7 Methods

### 7.1 Coupled Dipole Calculations

To theoretically model the meta rings discussed in the main text, we utilize a coupled point dipole model. Here, the i'th meta atom in the ring situated at position $r_{i}$ is approximated by a dipole source $p_{i}$, for which the scattered fields $E\left(r_{1}\right)$ at some position $r_{1}$ can be calculated by evaluating the Green's function $\overset{↔}{G}\;\left(r_{i},r_{1}\right)$ of the dipole in a homogeneous medium of refractive index n and permeability μ [50].

(6) $$\overset{↔}{G}\left(r_{i},r_{1}\right)=\left(\overset{↔}{I}+\frac{1}{k^{2}}\nabla \nabla \right)\frac{e^{ik|r_{i}-r_{0}|}}{4\pi |r_{i}-r_{0}|},$$

where $\overset{↔}{I}$ is the unit tensor and *k* the wavenumber in the medium. The scattered fields are then given by:

(7) $$E\left(r_{1}\right)=\omega^{2}\mu \mu_{0}\overset{↔}{G}\left(r_{i},r_{1}\right)\cdot p_{i},$$

where ω is the driving frequency and $\mu_{0}$ the permeability of free space. For the meta ring ensemble consisting of N coupled dipoles $\left\{p_{1}\text{…}p_{N}\right\}$ driven by an incident excitation field $E_{\mathrm{ext}}$, a solution of coupled dipoles is retrieved by solving the matrix equation

(8) $$\left[\begin{array}{c} p_{1}\\ \text{⋮}\\ p_{N} \end{array}\right]=M^{-1}\cdot \left[\begin{array}{c} E_{\mathrm{ext}}\left(r_{1}\right)\\ \text{⋮}\\ E_{\mathrm{ext}}\left(r_{N}\right) \end{array}\right],$$

where

(9) $$M_{i,j}=\delta_{i,j}{\left(\overset{↔}{\alpha_{i}}\right)}^{-1}-\left(1-\delta_{i,j}\right)\overset{↔}{G}\left(r_{i},r_{j}\right)$$

is the (3N×3N) coupling matrix of the system that described all mutual interactions between the dipoles in the ensemble. In this expression, $\overset{↔}{\alpha_{i}}$ is the dynamic polarizability tensor of the *i*'th particle.

## Author Contributions

**Nick Feldman:** conceptualization, writing – original draft, data curation, methodology, validation, investigation, writing – review and editing, visualization, formal analysis. **Nelson de Gaay Fortman:** software, methodology, investigation. **Arie J. den Boef:** writing – review and editing, validation, investigation. **Lyubov V. Amitonova:** funding acquisition, writing – review and editing, conceptualization, project administration, supervision, validation, investigation. **A. Femius Koenderink:** conceptualization, investigation, funding acquisition, writing – review and editing, validation, project administration, supervision.

## Supporting information


Supporting Information S1

## Data Availability

The data that support the findings of this study are available as a fully open source package containing data and source code from ZENODO (https://doi.org/10.5281/zenodo.20021254).
