# Supplementary material for: Wavefront Selective Modal Excitations for Optimally Informative Sensing in Fano‐Resonant Metasurfaces
Source: Nanophotonics. 2026 May 9;15(9):e70119. doi: 10.1002/nap2.70119 (PMC13642524; doi:10.1002/nap2.70119)
Supplement: Supplementary file 1 [file NAP2-15-e70119-s001.pdf]

Supplementary Information for "Wavefront selective modal excitations for optimally informative sensing in Fano-resonant metasurfaces"

Nick Feldman,[†,‡] Nelson de Gaay Fortman,[¶] Arie J. den Boef,[‡,§,‖] Lyubov V. Amitonova,[‡,§] and A. Femius Koenderink[*,†]

†*Department of Information in Matter and Center for Nanophotonics, AMOLF, Science Park 104, 1098 XG, Amsterdam, Netherlands*

‡*Advanced Research Center for Nanolithography (ARCNL), Science Park 106, 1098 XG Amsterdam, The Netherlands*

¶*Department of Information in Matter and Center for Nanophotonics, AMOLF, Science Park 104, 1098 XG, Amsterdam, The Netherlands*

§*Department of Physics and Astronomy, and LaserLaB, Vrije Universiteit, De Boelelaan 1081, 1081 HV Amsterdam, The Netherlands*

‖*ASML Netherlands B.V., De Run 6501, 5504 DR, Veldhoven, The Netherlands*

E-mail: f.koenderink@amolf.nl

Eigenmode profiles and mode indexing

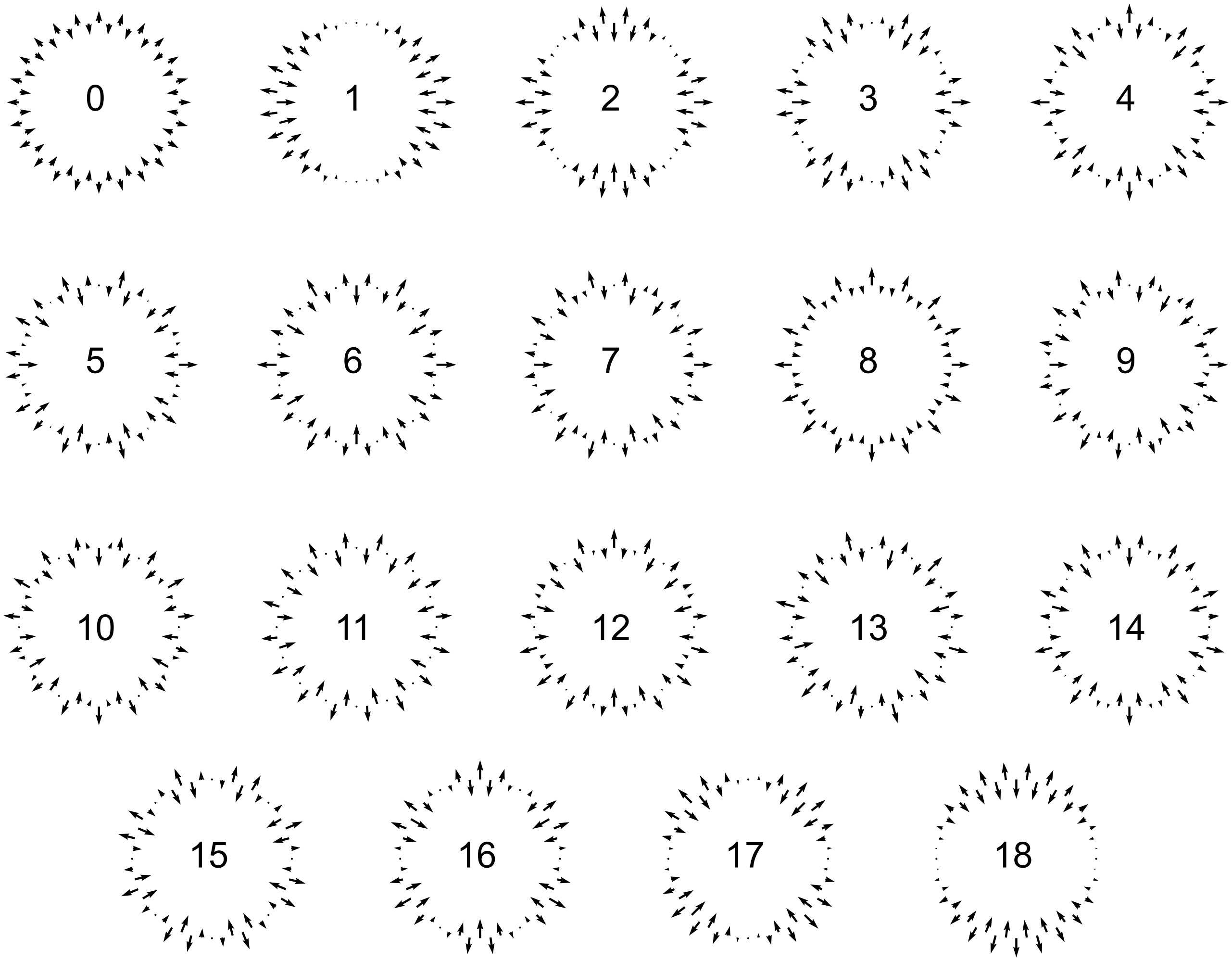

Figure S1: **Complete set of dominant eigenmode profiles and corresponding mode indices.**
